# Urolithiasis, Urinary Cancer, and Home Drinking Water Source in the United States Territory of Guam, 2006–2010

**DOI:** 10.3390/ijerph13060523

**Published:** 2016-05-24

**Authors:** Robert L. Haddock, David R. Olson, Lorraine Backer, Josephine Malilay

**Affiliations:** 1 Guam Department of Public Health and Social Services, 123 Chalan Kareta, Mangilao, GU 96913, USA, robhad@yahoo.com; 2 Centers for Disease Control and Prevention, National Center for Environmental Health, 4770 Buford Hwy., NE (Mail Stop F-60), Atlanta, GA 30341, USA; lbacker@cdc.gov (L.B.); jmalilay@cdc.gov (J.M.)

**Keywords:** urolithiasis, urinary cancer, municipal water, hard water, well water, calcium carbonate

## Abstract

We reviewed patient records with a first-listed diagnosis of urolithiasis—also known as urinary tract or kidney stone disease, nephrolithiasis—upon discharge from Guam’s sole civilian hospital during 2006 to 2010 and urinary cancer mortality records from the Guam Cancer Registry for 1970 to 2009 to determine the source of municipal water supplied to the patients’ residence. The objective was to investigate a possible relationship between the sources of municipal water supplied to Guam villages and the incidence of urolithiasis and urinary cancer. We analyzed hospital discharge diagnoses of urolithiasis or renal calculi by calculating the incidence of first-mentioned discharge for urolithiasis or renal calculi and comparing rates across demographic or geographic categories while adjusting by age, sex, and ethnicity/race. We reviewed cancer registry records of urinary cancer deaths by patient residence. The annual incidence of hospitalization for urolithiasis was 5.22 per 10,000. Rates adjusted for sex or age exhibited almost no change. The rate of 9.83 per 10,000 among Chamorros was significantly higher (*p* < 0.05) than the rates among any other ethnic group or race. When villages were grouped by water source, rates of patients discharged with a first-listed diagnosis of urolithiasis, adjusted for ethnicity/race, were similar for villages using either well water (5.44 per 10,000) or mixed source water (5.39 per 10,000), and significantly greater than the rate for villages using exclusively reservoir water (1.35 per 10,000). No statistically significant differences were found between the water source or village of residence and urinary cancer mortality. Some Guam residents living in villages served completely or partly by deep well water high in calcium carbonate may be at increased risk for urolithiasis compared with residents living in villages served by surface waters. Although the risk appears to be highest in villagers of Chamorro ethnicity, residents should be aware of other contributing risk factors and steps to take to avoid developing this health problem.

## 1. Introduction

The prevalence and incidence of urolithiasis—also known as urinary tract or kidney stone disease, nephrolithiasis—have been rising worldwide in recent decades. In the United States (U.S.), the prevalence of kidney stone disease in adults aged 20–74 years increased from 3.8% in 1976–1980 to 5.2% in 1988–1994 [1]. According to the 2007–2010 National Health and Nutrition Examination Survey, 8.8% of U.S. men and women were found to have to kidney stone disease [2]. Elevated incidence rates have been observed in some regions of the U.S. (1116 per 100,000 in 2000) and Japan (93 per 100,000 in 1993) [3]. Race/ethnicity, age, and sex differences have been noted [1,2,3,4] along with comorbidities such as obesity and diabetes [2]. Changes in dietary calcium and protein, dehydration, and warm and humid climates are frequently associated with increased rates [1,5,6,7]. Water hardness from public water supply systems is thought to be related to urinary stone disease, largely due to public perception. However, studies have not noted a link between water hardness or water quality and the incidence of urinary stone formation [8,9,10]. One study identified an increased risk of urinary stone disease and consumption of water from private wells [11]. To further explore this perception, we examined the source of municipal water and diagnoses of urolithiasis and urinary cancer among village residents in the United States Territory of Guam, where natural geologic features lead to differentiated sources of water for the local municipal water systems.

The southernmost island of the Marianas Archipelago in the Western Pacific, Guam is the largest (approximately 212 square miles), tallest (1332 feet), and most populous (159,358 residents in 2010) island of Micronesia [12]. The northern half of the island was formed by highly permeable uplifted ancient coral reefs, resulting in no rivers, streams, permanent lakes or ponds. The southern half of the island was formed by volcanic activity, leading to the formation of rivers and streams from the relative impermeability of the soil [13,14]. In contrast to several neighboring islands that are part of low-lying coral atolls, the elevation of the island makes it less likely to be affected by rising sea levels. The annual rainfall averages about 85 inches. Monthly precipitation is about 8–14 in during the rainy season occurring from July to November, and about 2–4 in during the dry season, usually from January to May [15]. Daily temperatures range from 76 to 87 °F, with a relative humidity of 75% to 85% during the rainy season [15]. A change in the economy from rural subsistence to employment by the public sector and private enterprise after World War II led to shifts in dietary patterns. Locally grown fruits and vegetables, meats, and fish have been replaced in part by a diet of imported goods containing more sugar, canned foods, sweetened drinks, and other convenience food products [16]. 

Municipal water supplied to the northern and central villages of Guam originates from numerous deep wells passing through the coral formation to a fresh water lens floating above infiltrated seawater. Municipal water supplied to the three southern villages of Guam originates from a single stream-fed reservoir (the Ugum Reservoir located in the municipality of Talofofo). The water provided to all other villages originates from wells or is a mixture of well and surface water [17].

A study was initiated to determine relationships between the source of municipal water provided to civilian residents of Guam and a diagnosis of renal stones. 

## 2. Methods 

We used the U.S. Census Bureau’s 2010 Guam Summary File to obtain population estimates for villages and Guam overall. Census values were categorized by ethnic origin or race and by age and sex. We reviewed patient records with a first-listed discharge diagnosis of urolithiasis or renal calculi (ICD-9-CM codes 592.0–592.9) upon discharge from Guam’s sole civilian hospital during the study years of 2006 to 2010. Comparable records from the military hospital on Guam were unavailable. The actual residence of patients was confirmed by reviewing individual patient records if the computer-listed address contained only a mailing address. The type of water provided to the residence of these patients was obtained from a government report [17]. Data were extracted from hospital records and mortality records from the Guam Cancer Registry. The Guam Department of Public Health and Social Services does not seek approval for human subjects review for studies from aggregated data with no patient contact. The Centers for Disease Control (CDC) and Prevention’s National Center for Environmental Health/Agency for Toxic Substances and Disease Registry determined that the CDC’s role is “not human subjects research” in support of the Guam Department of Public Health and Social Services (internal communications, 14 February 2014). Further, CDC authors have no linkages to identifying information on data abstracted from secondary sources by the Guam Department of Public Health and Social Services (internal communications, 25 February 2014). 

We calculated rates using cases of urolithiasis as the numerator and the appropriate census population estimate as the denominator. To compare rates across demographic or geographic categories, we assigned Bernoulli outcomes across the census (1 = urolithiasis, 0 = not). Assuming independence, the sum of Bernoulli outcomes in any category is binomial. Since each binomial outcome has a large value for n, we tested for differences in rates (proportions) using the normal distribution as an approximation and adjusted for multiple comparisons as needed. We also adjusted rates by age, sex, and ethnicity/race using the overall Guam population as the standard. Statistical significance for differences was set at 0.05 (with a Bonferroni correction where appropriate).

Although consumption of hard water is not known to be a risk factor for kidney cancer, we also reviewed records from the Guam Cancer Registry for 1970 to 2009 to identify cases of death due to urinary cancers and to determine the residence village of these cases [18].

## 3. Results

During 2006–2010, 416 patients were discharged from Guam Memorial Hospital with a first-listed diagnosis of urolithiasis or renal calculi, yielding an annual rate of 5.22 per 10,000 (Table 1). Rates for males and females were similar, with 5.17 per 10,000 and 5.27 per 10,000, respectively. The rate for persons aged 30–49 was highest (9.62 per 10,000), but was not significantly different from the rate for persons aged 50 or greater (7.94 per 10,000). The rate for persons aged 18–29 was significantly smaller at 4.34 per 10,000, while the rate for persons aged less than 18 was significantly smaller still at 0.19 per 10,000. The rate of 9.83 per 10,000 among Chamorros (defined as the indigenous people of Guam) was significantly higher than the rates among any other ethnicity/race. The rate among Micronesians (defined as Pacific Islanders from the Micronesian region including the Federated States of Micronesia, Republic of Belau, and the Republic of the Marshall Islands) was significantly higher than the rate for the “Other” ethnicity/race category. The rates for Asians, Filipinos, Micronesians, and whites were not significantly different.

Patients identifying as Chamorros accounted for 70% of the cases of urolithiasis. Among Chamorros, the annual rate for females of 10.6 per 10,000 was greater than the rate for males of 9.07 per 10,000 (see Table 2), but this difference was not statistically significant. Among Chamorros, the rate for persons aged 30–49 was highest (20.36 per 10,000). This rate was significantly higher than the rates for the other age groups. The rate for persons aged 50 or greater (12.86 per 10,000) was not significantly different from the rate for persons aged 18–29 (9.25 per 10,000). The rate for persons aged less than 18 was significantly smaller at 0.48 per 10,000.

**Figure 1:**
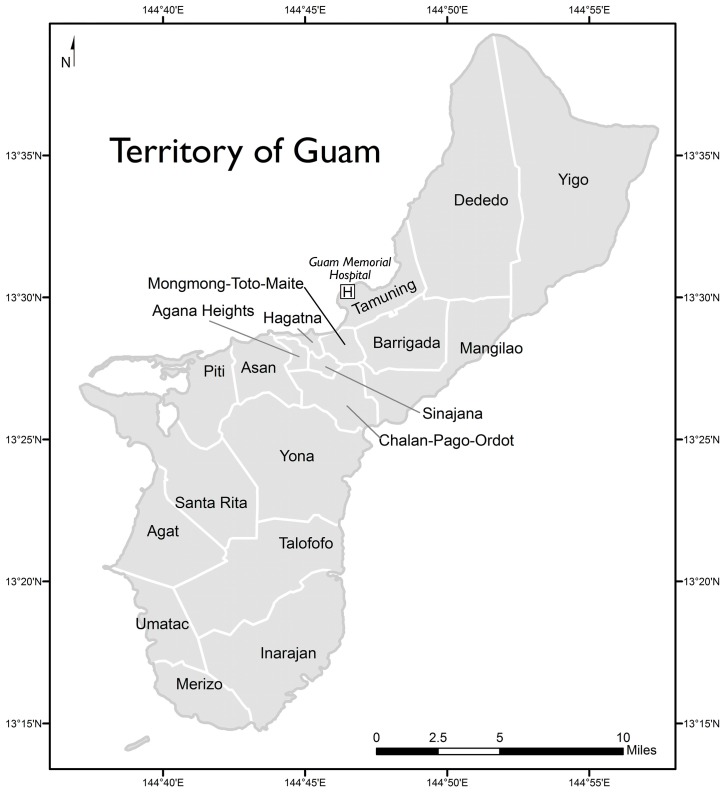
Territory of Guam with locations of study hospital and villages.

The locations of villages on the island of Guam are shown in Figure 1. Rates of patients discharged with a first-listed diagnosis of urolithiasis or renal calculi by village are shown in Table 3. Rates adjusted for sex or age exhibited almost no change (data not shown). However, adjusting rates for ethnicity/race produced noticeable changes and these rates are also shown in Table 3. In the table, villages are grouped by water source. Pairwise comparisons of villages yielded the following statistically significant differences in rates adjusted for ethnicity/race: Yigo had the highest rate of 13.86 per 10,000, which was greater than the rates for all other villages except for Agat, Piti, Sinajana, and Tamuning. Tamuning had the second highest rate of 10.89 per 10,000, which was greater than the rates for Agana Heights, Chalan Pago-Ordot, Dededo, Hagatna, Inarajan, Mangilao, Merizo, Talofofo, and Umatac. The rate of 0 for Hagatna was smaller than the rates for Agat, Barrigada, Chalan Pago-Ordot, Dededo, Mangilao, Mongmong-Toto-Maite, and Santa Rita. All other pairwise comparisons of rates were not significantly different.

When villages are grouped by water source, rates of patients discharged with a first-listed diagnosis of urolithiasis or renal calculi adjusted for ethnicity/race are similar for villages using either well water or mixed source water. These rates are significantly greater than the rate for villages using exclusively reservoir water (Table 3).

There were no statistically significant differences between water source or village of residence and urinary cancer mortality (data not shown).

## 4. Discussion

To our knowledge, this study is the first to estimate the incidence of urolithiasis in Guam. We have characterized this incidence by sex, age, and ethnicity or race, using the hospital discharge diagnosis to identify case patients and the village of residence to determine drinking water sources for case patients’ households. During 2006–2010, 416 patients treated and discharged from Guam Memorial Hospital had a first-listed diagnosis of urolithiasis or renal calculi (ICD-9-CM 592.0–592.9). The average annual incidence of first-listed diagnoses of urinary calculus for discharges for Guam was 5.5 per 10,000, an estimated 22% higher than the U.S. rate of 4.5 per 10,000 [19]. Rates adjusted for sex or age exhibited almost no change. The hospitalization rate for urolithiasis among villages supplied with water wholly or partially from deep wells was about twice that of villages supplied with municipal water exclusively from a river-fed reservoir. The rate in the mostly southern reservoir-served villages (2.85 per 10,000) was an estimated 37% less than the U.S. rate (4.5 per 10,000) [19]. No statistically significant differences were observed between water sources and urinary cancer mortality.

Although we were unable to analyze the variation in exposure by household or the length of exposure at the village of residence, the unique geology of the island provided the basis for a “natural” experiment in the effects that municipal drinking water supplies from surface (reservoir) water, ground (well) water, and a mixture of surface and ground water may have on populations served from these sources. The three southern Guam villages supplied with municipal water from a reservoir are largely rural, more traditional in their way of life, and populated by residents primarily of Chamorro ethnicity [12]. These villages exhibited low rates of urolithiasis, despite their residents being primarily of Chamorro ethnicity. However, their ethnicity-adjusted rates are even lower (1.03–1.62 hospital discharges per 10,000 estimated civilian village population per year), consistent with the result that persons with Chamorro ethnicity had the highest rate among the major ethnic groups present on the island for the territory as a whole (9.83 hospital discharges per 10,000 estimated civilian village population per year). To further assess the pattern of urolithiasis incidence in Guam, particularly among Chamorros, additional information about known or suspected risk factors, such as obesity and high protein or fat intake, hypertension, or chronic kidney disease, and protective factors, such as adequate fluid intake, would be needed from patients [20]. A nutrition study among 127 volunteer adults in Guam found that, overall, 66% of study subjects were overweight (body mass index > 25) or obese [21]. Further, the risk of developing kidney stones may vary by type of diet; for example, increased oxalate, sodium, and animal protein consumption may promote stone formation. In contrast, a reduction of sodium and animal protein and normal calcium intake attenuates stone formation when patients have recurrent hypercalciuric stones [22]. Warm temperatures and high humidity characteristic of a tropical climate may necessitate increased appropriate and adequate fluid intake.

It is also possible that the differences in urolithiasis rates observed in the villages are associated with other environmental factors, including characteristics of drinking water (in addition to differences in calcium carbonate) derived from wells passing through coral reefs compared with drinking water derived from surface waters flowing over volcanic rock. Also, local land use (e.g., development, agriculture) would determine which naturally occurring or anthropogenic chemicals either percolate through the coral reefs to the ground water in northern Guam or run off into the surface rivers and lakes overlying the volcanic rock in southern Guam.

In 2010, the testing of water from Guam wells found a calcium carbonate (CaCO_3_) content ranging from 172 to 610 ppm, from the Fena Reservoir at levels of 90 to 124 ppm, and from the Ugum Reservoir at 40 to 92 ppm [17]. Drinking hard water has been associated with an increased risk of developing calcium urinary stones in one study [23], but not in others [8,9,10]. In one study, drinking water from private wells as opposed to municipal water had an estimated relative risk of 1.5 (*p* < 0.01) for urinary stone formation; the effect could not be associated with calcium, magnesium or sodium concentrations [11]. There may be another characteristic of or common contaminant in private well–derived drinking water that is not typically present in surface waters. As a comparison, most municipal drinking water supplies in the United States originate from rivers or surface waters collected in reservoirs [24]. Additionally, treating for hardness is not performed as the well water in Guam is not hard enough to warrant softening (personal communications, Guam Waterworks Authority, 3 May 2016).

Several limitations were associated with this ecologic study on the basis of hospital discharge diagnosis and village of residence. Specific drinking water sources (deep well, surface water, or mixed) could not always be assigned to individual case patients. Moreover, consumption of bottled water, a primary source of drinking water reported by an estimated 77% of respondents in a survey on drinking water trends on the island, could not be evaluated [25]. CaCO_3_, Ca, Mg, and HCO_3_ data for village wells were unavailable. Also, we did not have information to characterize other risk factors for urolithiasis, such as diet, coffee and tea consumption, extreme temperatures, urine calcium excretion, stone composition, geographical variation, and obesity [3,26,27,28]. Although an estimated 17% of the island’s population is eligible to be treated at the only other hospital at the time of this study, a military facility, we collected data solely from the civilian hospital with the assumption that their patients would have had the most consistent history of exposure to the local water supply, whereas frequently rotating tours of duty would have limited exposures among military personnel and their families to Government of Guam–supplied drinking water for extended periods [29]. Finally, we acknowledge the possibility of misclassification because chart reviews were not undertaken to confirm accuracy. Nevertheless, results of this study pose further hypotheses for exploring potential risk factors for urolithiasis in the western Pacific. 

## 5. Conclusions

Although no difference was observed with respect to kidney cancer, it appears that Guam residents living in villages served completely or partly by deep well water high in calcium carbonate may be at increased risk for urolithiasis compared with residents living in villages served by surface waters. However, this risk may be mitigated by ethnicity; thus, residents of these villages should be aware of other contributing risk factors and steps that they can take to avoid developing this health problem.

## Figures and Tables

**Table 1:** Incidence of first-mentioned discharge for urolithiasis by age, sex and ethnicity/race, Guam Memorial Hospital, 2006–2010.

	Population ^a^	Discharges	Rate ^b^
Age			
<18	52,312	5	0.19
18–29	28,539	62	4.34
30–49	44,481	214	9.62
50+	34,026	135	7.94
Sex			
Female	77,790	205	5.27
Male	81,568	211	5.17
Ethnicity/Race			
Asian	9437	12	2.54
Chamorro	59,381	292	9.83
Filipino	41,944	53	2.53
Micronesian	18,044	35	3.88
White	11,321	22	3.89
Other	4302	2	0.93
Guam Total	159,358	416	5.22

^a^ Estimated civilian population from the 2010 census; ^b^ Rate = Hospital discharges per 10,000 estimated civilian population per year.

**Table 2:** Incidence of first-mentioned discharge for urolithiasis by age and sex among patients identifying as Chamorros, Guam Memorial Hospital, 2006–2010.

	Population ^a^	Discharges	Rate ^b^
Age			
<18	21,007	5	0.48
18–29	10,157	47	9.25
30–49	15,622	159	20.36
50+	12,595	81	12.86
Sex			
Female	29,619	157	10.60
Male	29,762	135	9.07
Chamorro Total	59,381	292	9.83

^a^ Estimated civilian population from the 2010 census; ^b^ Rate = Hospital discharges per 10,000 estimated civilian population per year.

**Table 3:** Incidence of first-mentioned discharge for urolithiasis by village of residence, Guam Memorial Hospital, 2006–2010.

Village	Population ^a^	Discharges	Rate ^b^	Adj. Rate ^c^
Water from Wells				
Yigo	20,539	107	10.42	13.86
Dededo	44,943	47	2.09	2.48
Tamuning	19,685	70	7.11	10.89
Mangilao	15,191	37	4.87	4.39
Barrigada	8875	27	6.08	5.30
Chalan Pago-Ordot	6822	21	6.15	4.13
Agana Heights	3808	8	4.20	3.00
Yona	6480	13	4.01	4.11
Talofofo	3050	3	1.97	1.11
Total	129,393	333	5.15	5.44
Mixed-Source Water (Fena Reservoir + Wells)				
Hagatna	1051	0	0.00	0.00
Agat	4917	16	6.51	7.37
Santa Rita	6084	19	6.25	5.81
Asan-Maina	2137	8	7.49	4.77
Mongmong-Toto-Maite	6825	16	4.69	4.55
Piti	1454	5	6.88	7.42
Sinajana	2592	9	6.94	8.06
Total	25,060	73	5.83	5.39
Ugum Reservoir Water				
Umatac	782	1	2.56	1.28
Merizo	1850	2	2.16	1.03
Inarajan	2273	4	3.52	1.62
Total	4905	7	2.85	1.35
Guam Total	159,358	416	5.22	

^a^ Estimated civilian population of villages from the 2010 census; ^b^ Rate = Hospital discharges per 10,000 estimated civilian village population per year; ^c^ Adjusted for ethnicity/race using overall Guam population as the standard.

## Abbreviations

The following abbreviations are used in this manuscript:

U.S.	United States
CDC	Centers for Disease Control and Prevention
GMHA	Guam Memorial Hospital Authority
